# Comparative assessment of the relationship between coliform bacteria and water geochemistry in surface and ground water systems

**DOI:** 10.1371/journal.pone.0257715

**Published:** 2021-09-21

**Authors:** Simon Appah Aram, Benjamin M. Saalidong, Patrick Osei Lartey

**Affiliations:** 1 Research Center for Smart Mine and Intelligent Equipment, Taiyuan University of Technology, Taiyuan, People’s Republic of China; 2 College of Safety and Emergency Management Engineering, Taiyuan University of Technology, Taiyuan, People’s Republic of China; 3 Department of Geosciences, Taiyuan University of Technology, Taiyuan, People’s Republic of China; 4 Ministry of Education Key Laboratory of Interface and Engineering in Advanced Materials, Research Center of Advanced Materials Science and Technology, Taiyuan University of Technology, Taiyuan, People’s Republic of China; Kaohsiung Medical University, TAIWAN

## Abstract

The occurrence of pollution indicator bacteria (total and faecal coliform) has been used as a sanitary parameter for evaluating the quality of drinking water. It is known that these indicators are associated with disease causing organisms which are of great concern to public health. This study assessed the relationship between coliform bacteria and water geochemistry in surface and ground water systems in the Tarkwa mining area using logistic regression models. In surface water sources, higher values of chloride (OR = 0.891, p<005), phosphates (OR = 0.452, p<0.05), pH (OR = 0.174, p<0.05) and zinc (OR = 0.001, p<0.05) were associated with lower odds of faecal coliform contamination. In groundwater sources, higher values of phosphates (OR = 0.043, p<0.001), total dissolved solids (OR = 0.858, p<0.05), turbidity (OR = 0.996, p<0.05) and nickel (OR = 6.09E-07, p<0.05) implied non-contamination by faecal coliform. However, higher values of electrical conductivity (OR = 1.097, p<0.05), nitrates (OR = 1.191, p<0.05) and total suspended solids (OR = 1.023, p<0.05) were associated with higher odds of faecal coliform contamination of groundwater sources. Nitrates and total suspended solids, in this case, were completely mediated by the heavy metals. For total coliform in surface water systems, higher values of magnesium (OR = 1.070, p<0.05) was associated with higher odds of total coliform contamination while higher values of phosphates (OR = 0.968, p<0.05) was associated with lower odds of total coliform contamination although the presence of heavy metals completely mediated these relationships. For ground water systems, higher values of pH (OR = 0.083, p<0.05), phosphates (OR = 0.092, p<0.05), turbidity (OR = 0.950, p<0.05) and chloride (OR = 0.860, p<0.05) were associated with lower odds of total coliform contamination. However, higher values of total suspended solids (OR = 1.054, p<0.05) and nitrates (OR = 1.069, p<0.05) implied contamination of total coliform in ground water sources. The relationship between nitrates and total coliform were mediated by the heavy metals. This study establishes the need to monitor, manage and remediate surface and ground water sources for potential disease causing microbes in ways that takes into consideration the factors that create different conditions in the two water systems. This study validates the usefulness of statistical models as tools for preventing surface and ground water contamination.

## Introduction

Tremendous increase in human activities such as urbanization and industrialization, have disturbed the balance of the environment considerably, and aquatic systems are no exception to this. This phenomenon has led to the threatening of microbial quality of waters by contaminating it with untreated domestic waste waters and other industrial discharges [1]. This has led to the presence of coliform bacteria in water bodies which in many cases exceed the recommended limit for domestic purposes such as drinking, recreation or irrigation of crops eaten raw. Water plays an important role in the spread of innumerable diseases causing endemic and epidemic outbreaks in many parts of the world. It is reported that over 250 million people get infected by waterborne diseases each year and about 10 million of them die [2]. Also 30,000 people have been reported to die every day in developing countries as a result of consumption of unsanitary water [3].

Many human diseases such as cholera, typhoid fever, dysentery and gastroenteritis are believed to be caused and spread by bacteria from the faeces of sick persons which contaminates food and water used by other healthy people. Coliform bacteria have become the traditional and universal microbiological indicators of water quality [4, 5]. The occurrence of pollution indicator bacteria (total and faecal coliform) has also been used as a sanitary parameter for evaluating the quality of drinking water [5]. It is known that these indicators are associated with disease causing organisms which are of great concern to public health. Potable and safe water supply in some parts of the world have become a problem due to their role in carrying bacteria to consumers. Coliform bacteria have been used as the indicator tool to measure the occurrence and intensity of faecal contamination in natural water for years. Bacteriological assessment particularly for coliforms are the indicators of contamination by faecal matters and are therefore routinely carried out by many public health officers to ascertain the quality and potability of water to ensure prevention of further dissemination of pathogens.

Reporting of water quality conditions is often untimely and inaccurate because monitoring programs might not be able to monitor these water bodies regularly as needed owing to financial constraints. In addition, time constraints could also impose monitoring challenges due to the fact that laboratory analysis of faecal coliform bacteria requires a 24-hour incubation period [6]. Due to these challenges, there has been the need to develop models that correlate pollutant concentrations with some more easily measurable factors [7]. Statistical models can also be used to determine preliminary probability distributions of impairment that can help direct monitoring efforts and reduce the quantity of monitoring data, and it can also be used to assess future quality situations [7]. Estimating water contamination through statistical modeling for an indicator organism such as coliform bacteria can be useful and could be a standard means to estimate water pollution.

The goal of this study is to create a statistical model for faecal and total coliform bacteria in water bodies around a mining environment and predict coliform levels from physiochemical parameters and heavy metal concentrations using generalized linear regression models. This will help to eliminate the over-reliance on water quality sampling. Total coliform and faecal coliform have been chosen for this study mainly because of their faecal and environmental origin and could also be monitored as an alternative for *E*. *coli* and as such have been used universally as microbiological indicators of water quality.

## Materials and methods

### Study area

**Fig 1:**
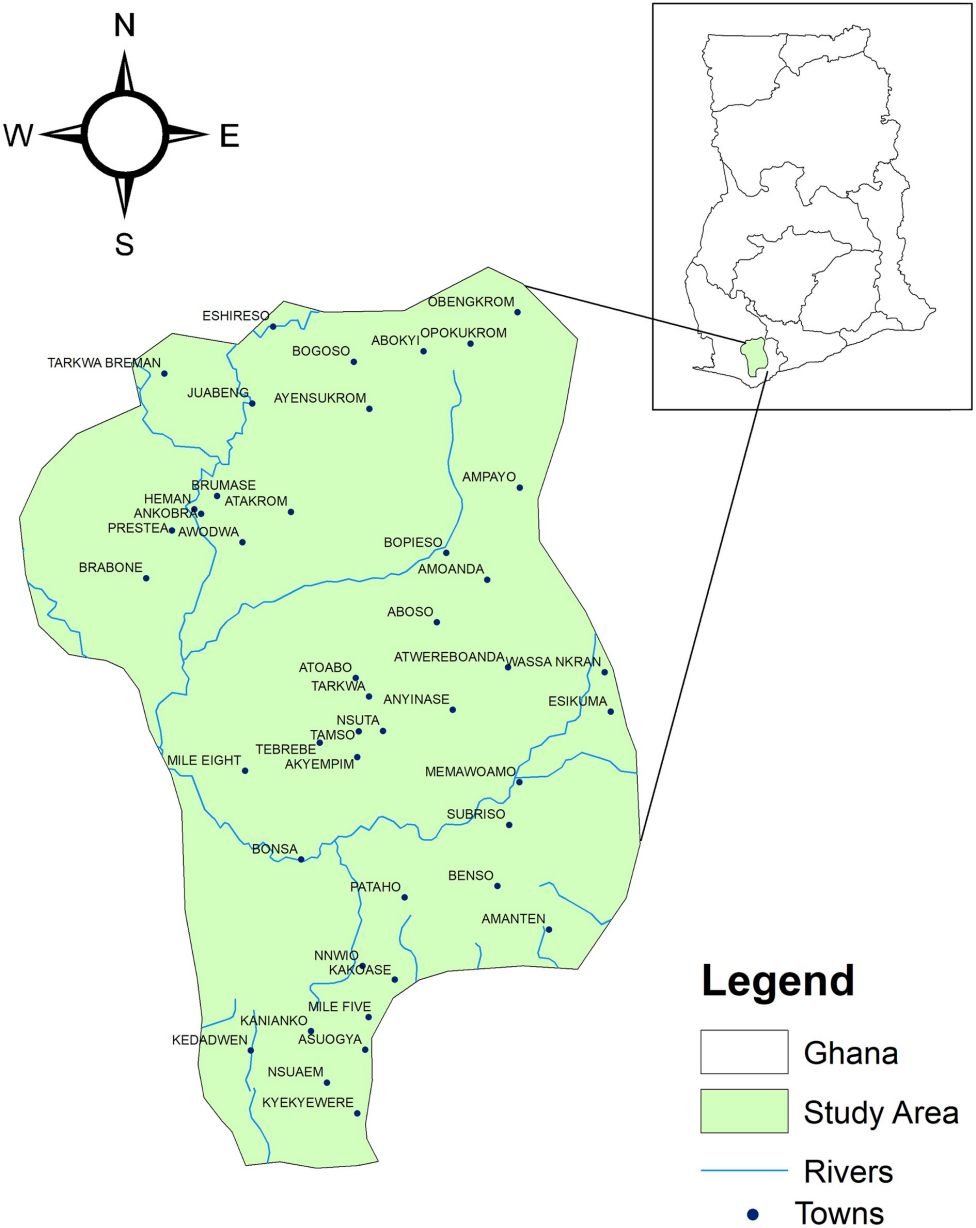
Map of the study area.

The data was taken from the Tarkwa mining area in the Western Region of Ghana as shown in Fig 1. The area is located between latitudes 4° 0ʹ 0″ N and 5° 40ʹ 0″ N and longitudes 1° 45ʹ 0″ W and 2° 1ʹ 0″ W. A total of 100 ground water and 132 surface water locations were selected for this study. Sampling was done between January 2019 and December 2019.

The domestic and commercial water supply systems in the area mainly consist of boreholes hand-dug wells, streams and rivers. The majority of these water supply systems serve as a source of drinking water for nearby communities. The average well depth in the area is 35.4 m. Borehole yields range between 0.4 m^3^/h and 18 m^3^/h with an average of 2.4 m^3^/h [8]. The Bonsa, Huni and Ankobra Rivers and their tributaries are the main sources of recharge for nearby streams and ground water [8]. The quality of the water supply systems in the area is highly affected by mine contaminants and mining-related activities, leakage from underground storage tanks, improper waste disposal and agrochemicals from agricultural fields [8].

### Data description

A total of 19 parameters, which include 11 physicochemical parameters; pH, electrical conductivity (EC), turbidity, total dissolved solids (TDS), total suspended solids (TSS), magnesium, nitrates, chloride, calcium, phosphates and dissolved oxygen (DO) and 8 heavy metals; zinc, iron, manganese, nickel, chromium, copper, lead and cadmium from surface and ground water systems were used for this study. The selection of these parameters to predict bacteria contamination was based on several factors; prior knowledge of the explanatory variables’ relationships with coliform bacteria and previous findings in literature concerning factors that influence microbiological organisms. Some parameters were also chosen based on their data availability and significance. Total coliform and faecal coliform were the focus variables of this study. The impact of physicochemical and heavy metal concentrations on the likelihood of contamination by total coliform and faecal coliform was analyzed using odds ratios (ORs), established by nested binary logistic regression models (generalized linear models).

### Nested binary logistic regression model

The impact of physicochemical parameters and heavy metal concentrations on the likelihood of contamination by total coliform and faecal coliform of ground and surface waters was examined using nested binary logistic regression models. Logistic regression allows the model to be related to the response variable via a link function and by allowing the magnitude of the variance of each measurement to be a function of its predicted value under the assumption of binary response (contaminated/ uncontaminated) [9]. Via the link function, there are several potential alternatives: the logit model, probit model, negative log-log and complementary log-log model. Both logit and probit link functions have the same property which is the probability that an observation in a specified category of a binary outcome variable (contaminated/ uncontaminated) has the same probability of approaching 0 as well as approaching 1 (50% uncontaminated, 50% contaminated). If the observations of a binary outcome have an asymmetrical success of probability, that is, fewer 0s than 1s or more 0s than 1s, then the link function complementary log-log or negative log-log is chosen respectively.

In this study, 82.58% of surface water and 71% of groundwater sources had total coliform while 54.55% of surface water and 54% of ground water locations had faecal coliform. For this reason the negative log-log link function was appropriate for modelling total coliform and faecal coliform contamination. The odds ratios (OR) were built in a nested model starting from the physicochemical model and the heavy metals model. All statistical analyses were performed using Stata 15 (StataCorp, College Station, Texas) SE software at a Statistical significance of 0.05 and at a confidence interval of 95%.

### Water sampling

The sampling methods followed the protocols developed by [10]. Sampling bottles were pre-washed with detergent and rinsed with 10% hydrochloric acid and double-distilled water prior to sampling. At each of the sampling locations, the bottles were rinsed three times with the water to be collected to reduce or completely eliminate any contaminations that might be introduced. Surface water was sampled by gently lowering the sample bottle horizontally into the water with the mouth of the bottle directed upstream, taking reasonable measures to avoid suspended/floating debris. Thus, surface water samples were collected at the subsurface in order to avoid the colloidal layer as this can influence the concentration of certain parameters. Personnel entry into the water body was minimized as much as possible. At each sampling location, 1000 ml of water was collected using two 500 ml transparent plastic bottles, which were placed in an opaque material (black polyethylene bag), tied and finally kept in a cooler box. This procedure averts microbial growth, flocculation and reduce any adsorption on container surfaces, processes which could affect the results. Water from the community boreholes was collected at the faucet after it had been pumped for a while to obtain a steady flow before sampling. This was to be sure that the water been collected is freshly abstracted from the bore.

#### Field analysis

The following parameters were measured *in situ*, using the AQuanta multi-parameter water quality meter (Hydrolab Corporation, USA) during the sampling: pH, conductivity, dissolved oxygen and turbidity. Most calibrations were conducted in the field at the sample site. The pH probe was calibrated with pH 7 and 10 buffer solutions on the day of sampling.

#### Laboratory analysis

All laboratory tests were conducted in accordance with “Standard Methods for the Examination of Water and Wastewater” of the American Public Health Association, 1998 Edition. Homogenized subsamples were filtered, and acid-digested following the USEPA protocol 2002 [10] and analyzed for total iron, manganese, copper, lead, chromium, nickel and zinc using flame atomic absorption spectroscopy (AAS Shimadzu model 6401F) following USEPA protocol 2007 [10]. Unprocessed water samples were also analyzed for total dissolved solids, total suspended solids, chloride, sulphate, nitrates, phosphates, magnesium and calcium. Faecal coliform and total coliform were also determined by the membrane filtration technique.

## Results

### Summary statistics of surface water and groundwater biological and heavy metal contaminants

Summary statistics of surface water and groundwater parameters are presented in Tables 1 and 2. For ground water locations, faecal coliform and total coliform were detected in 54 and 71 locations respectively. Additionally, faecal coliform and total coliform bacteria were detected in 72 and 109 of surface water locations. Minimum faecal and total coliform for both ground and surface water locations were 0 cfu/100ml however, maximum numbers recorded varied across water system type. Surface water recorded maximum faecal and total coliform of 870 and 35620 cfu/100ml respectively while groundwater recorded 8220 cfu/100ml for faecal coliform and 41100 cfu/100ml for total coliform.

**Table 1:** Descriptive statistics of surface water samples (n = 132).

Variable	Mean	Std. Dev.	Min	Max
pH	7.004848	0.819534	4.16	9.95
EC	304.4394	182.9038	39	821
TDS	199.5076	124.4572	14	542
Turbidity	89.73712	307.7058	0.13	3180
TSS	190.6818	894.5858	3.0	7300
Calcium	25.36409	21.23085	0.01	100
Magnesium	6.740227	5.125704	0.01	31
Phosphates	7.52197	11.97199	0.1	55.2
Chloride	10.02318	18.34699	0.0	165.39
DO	2.259091	0.86131	0.11	5.16
Zinc	0.101742	0.165453	0.01	0.8
Copper	0.385606	0.752845	0.0	5
Nickel	0.338258	1.459486	0.01	16.5
Lead	0.001289	0.002158	0.0	0.005
Manganese	0.009614	0.023528	0.001	0.25
Chromium	0.311212	0.542165	0.001	2.7

Zinc, Copper, Nickel, Lead, Manganese, Chromium, TDS, TSS, calcium, magnesium, phosphates, chloride and DO were measured in mg/L. EC and turbidity were measured in *μ*S/cm and NTU respectively.

**Table 2:** Descriptive statistics of ground water samples (n = 100).

Variable	Mean	Std. Dev.	Min	Max
pH	6.737	0.663078	5.24	7.85
EC	281.37	168.2184	35.0	751
Turbidity	61.3464	220.1067	0.01	1540
TDS	185.03	118.7351	14	682
TSS	72.7	202.3992	1.0	1310
Magnesium	5.7882	6.17024	0.01	40.3
Nitrates	9.5298	15.54474	0.0	86.2
Chloride	8.1751	14.47615	0.01	115.97
Phosphates	5.8249	10.27773	o.05	39.7
Zinc	0.1306	0.266886	0.01	2.14
Iron	0.0555	0.140212	0.01	1.3
Manganese	0.01702	0.020071	0.001	0.098
Nickel	0.1359	0.209834	0.01	1.38
Chromium	0.27194	0.338674	0.001	1.62
Copper	0.5669	0.854977	0.0	2.95

Zinc, Copper, Nickel, Lead, Manganese, Chromium, TDS, TSS, magnesium, nitrates, phosphates and chloride were measured in mg/L. EC and turbidity were measured in *μ*S/cm and NTU respectively.

For surface water sources, zinc and lead concentrations were below the WHO guideline of 5mg/L and 0.01mg/L respectively for drinking water. For surface water locations, 93% and 97% recorded copper and manganese concentrations below the acceptable thresholds of 2.0mg/L and 04mg/L respectively. More than 50% of the sampled locations recorded nickel and chromium values higher than their respective acceptable limits which according to the WHO are highly carcinogenic if ingested in higher quantities.

Ground water sources recorded zinc and manganese concentrations well below their thresholds. Iron (62%) and copper (88%) had most of the locations recording values below their acceptable WHO limit of 0.3mg/L and 2mg/L respectively. Most of the locations (more than 50%) recorded nickel and chromium values higher than their respective acceptable limits as it was the case in surface water.

**Fig 2:**
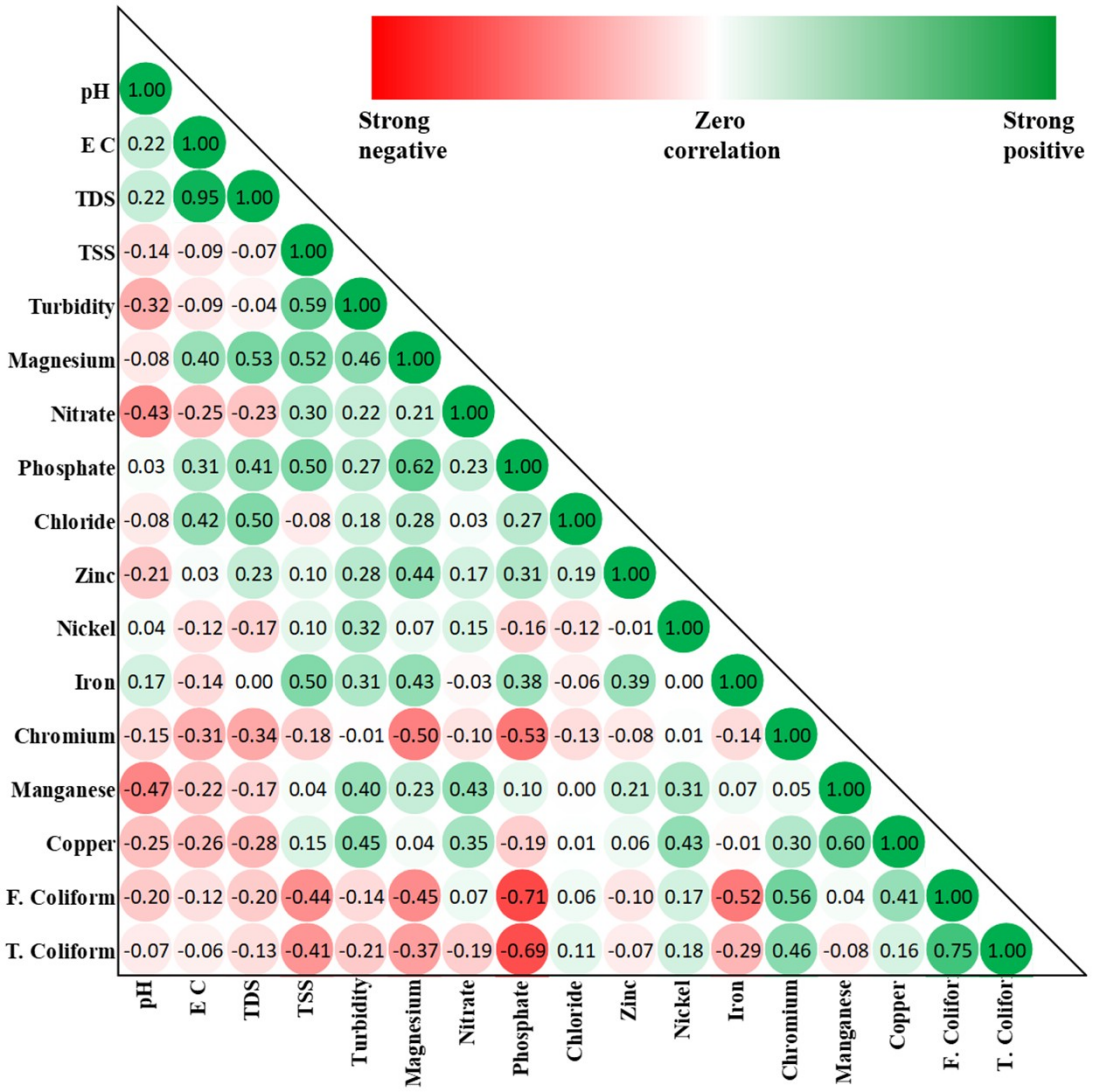
Pearson product moment correlation coefficients of log-transformed ground water data.

**Fig 3:**
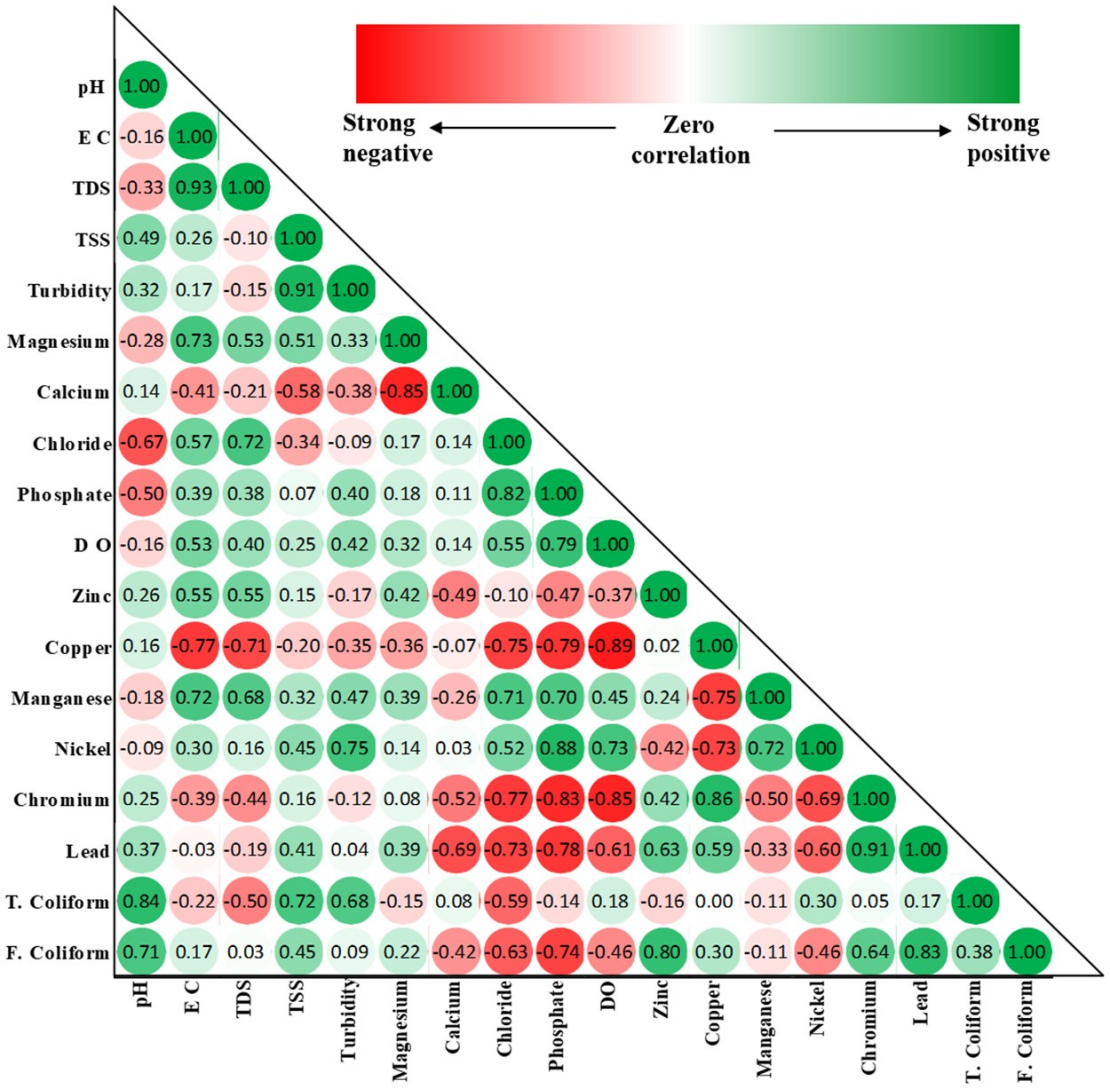
Pearson product moment correlation coefficients of log-transformed surface water data.

Figs 2 and 3 shows the correlation matrix for both faecal and total coliform bacteria in ground and surface water locations. Significant relationships were observed among some of the parameters at α-levels of 0.05.

### Bivariate relationship between faecal coliform and other surface and ground water parameters

Results for the bivariate analysis of faecal coliform are shown in Table 3. For the physicochemical parameters in surface water, higher values of magnesium (OR = 0.934, p<0.05), phosphates (OR = 0.601, p<0.001), chloride (OR = 0.960, p<0.05) and dissolved oxygen (OR = 0.128, p<0.001) were statistically associated with lower odds of faecal coliform contamination while higher values of total suspended solids (OR = 1.002, p<0.05) was associated with higher odds of faecal coliform contamination in surface water sources. For the selected heavy metals, zinc (OR = 0.006, p<0.001) and nickel (OR = 0.351, p<0.05) were statistically associated with lower odds of detecting faecal coliform. Higher values of lead (OR = 1.6E+202, p<0.001), copper (OR = 2.358, p<0.05) and chromium (OR = 5.426, p<0.05) were associated with higher odds of detecting faecal coliform.

**Table 3:** Negative log-log bivariate regression of water quality parameters and faecal coliform bacteria.

Parameters	Surface Water				Ground Water			
	OR	Robust SE	conf. Interval		OR	Robust SE	conf. Interval	
pH	0.914	0.141	0.675	1.237	0.960	0.186	0.657	1.402
Electrical Conductivity	1.000	0.001	0.999	1.002	1.000	0.001	0.999	1.002
Turbidity	1.000	0.001	0.999	1.002	1.000	0.001	0.999	1.002
Total Dissolved Solids	0.999	0.001	0.997	1.001	0.999	0.001	0.997	1.001
Total Suspended Solids	1.002*	0.001	1.000	1.004	1.000	0.001	0.998	1.001
Magnesium	0.934*	0.023	0.890	0.980	0.954*	0.020	0.915	0.995
Nitrates	1.001	0.005	0.991	1.011	1.023*	0.012	1.000	1.047
Phosphates	0.601*	0.046	0.517	0.698	0.120*	0.070	0.038	0.374
Chloride	0.960*	0.013	0.935	0.985	0.956*	0.019	0.918	0.994
Calcium	0.998	0.005	0.988	1.008	-	-	-	-
Dissolved Oxygen	0.128*	0.065	0.047	0.348	-	-	-	-
Zinc	0.006*	0.007	0.000	0.070	0.303	0.207	0.079	1.158
Iron	2.1338	1.352	0.616	7.385	16.896	29.094	0.578	493.732
Lead	1.6E+202*	1.6E+204	2.9E+117	8.8E+286	1.6E+124*	1.3E+126	8.39E+57	3.1E+190
Manganese	16.968	73.832	0.003	85774	3.13E-09*	1.73E-08	6.05E-14	0.000
Copper	2.358*	1.016	1.014	5.486	1.487	0.355	0.931	2.372
Nickel	0.351*	0.127	0.173	0.714	0.020*	0.018	0.003	0.122
Chromium	5.426*	4.247	1.171	25.156	3.250	2.173	0.876	12.052

For the physicochemical factors in ground water sources, higher values of magnesium (OR = 0.954, p<0.05), phosphates (OR = 0.120, p<0.001) and chloride (OR = 0.956, p<0.05) were associated with lower odds of faecal coliform contamination while higher values of nitrates (OR = 1.023, p<0.05) was associated with higher of odds of faecal coliform contamination. For the heavy metals, manganese (OR = 3.13E-09, p<0.001) and nickel (OR = 0.020, p<0.001) were associated with lower odds of detecting faecal coliform. Lead (OR = 1.6E+124, p<0.001) however was statistically related to higher odds of detecting faecal coliform.

### Bivariate relationship between total coliform and other surface and ground water parameters

Results for the bivariate analysis of total coliform are shown in Table 4. Of the physicochemical and heavy metals, higher values of magnesium (OR = 1.051, p<0.05) and manganese (OR = 2.61E+10, p<0.05) were statistically associated with higher odds of total coliform contamination in surface water sources.

**Table 4:** Negative log-log bivariate regression of water quality parameters and total coliform.

Parameters	Surface Water				Ground Water			
	OR	Robust SE	conf. Interval		OR	Robust SE	conf. Interval	
pH	0.883	0.127	0.666	1.170	0.741	0.136	0.516	1.063
Electrical Conductivity	1.001	0.001	1.000	1.002	0.999	0.001	0.998	1.001
Turbidity	1.000	0.000	1.000	1.001	1.000	0.001	0.999	1.001
Total Dissolved Solids	1.001	0.001	1.000	1.003	0.999	0.001	0.996	1.001
Total Suspended Solids	0.999	0.001	0.997	1.001	1.001	0.001	0.999	1.003
Magnesium	1.051*	0.022	1.008	1.096	0.950*	0.022	0.908	0.995
Nitrates	1.001	0.004	0.992	1.009	1.025*	0.011	1.004	1.046
Phosphates	1.002	0.009	0.985	1.020	0.301*	0.134	0.126	0.721
Chloride	0.988	0.009	0.970	1.006	0.951*	0.022	0.910	0.995
Zinc	2.600	1.801	0.669	10.104	0.629	0.239	0.298	1.326
Iron	0.945	0.435	0.383	2.330	31.880	57.769	0.914	1111.62
Cadmium	4.66E-66	6.08E-64	3.8E-177	5.72E+45	3.5E+177*	5.1E+179	1.60E+53	7.6E+301
Lead	2.01E-24	1.04E-22	1.50E-68	2.69E+20	1.04E+71*	6.08E+72	1.91E+21	5.7E+120
Manganese	2.61E+10*	3.10E+11	1.993	3.41E+20	0.001	0.004	4.29E-08	12.638
Copper	0.824	0.118	0.623	1.090	1.322	0.233	0.936	1.868
Nickel	1.125	0.074	0.989	1.281	0.163*	0.110	0.044	0.608
Chromium	0.719	0.127	0.509	1.016	1.892	0.969	0.693	5.163

For groundwater sources, higher values of magnesium (OR = 0.950, p<0.05), phosphates (OR = 0.301, p<0.05) and chloride (OR = 0.951, p<0.05) were associated with lower odds of detecting total coliform. Higher values of nitrates (OR = 1.025, p<0.05) however was associated with higher odds of detecting total coliform. Of the heavy metals, cadmium (OR = 3.5E+177, p<0.05) and lead (OR = 1.04E+71, p<0.05) were associated with detecting total coliform while nickel (OR = 0.163, p<0.001) was associated with odds of not detecting total coliform.

### Multivariate regression model of the relationship between faecal coliform, physicochemical and heavy metal concentrations in surface water

Table 5 is a nested model showing the multivariate relationship between faecal coliform, physicochemical and heavy metal concentrations in surface water. In the first model, which accounted for only the physicochemical parameters, pH (OR = 0.313, p<0.05), chloride (OR = 0.893, p<0.05) and phosphates (OR = 0.542, p<0.001) were statistically associated with lower odds of detecting faecal coliform in surface water locations. When the concentrations of heavy metals were adjusted for in the second model, the relationship between pH, chloride and phosphates, and the odds of detecting faecal coliform was persistent. Of the four heavy metals, only zinc (OR = 0.0001, p<0.05) was statistically associated with odds (lower) of detecting faecal coliform.

**Table 5:** Multivariate negative log–log regression model predicting detection of faecal coliform in surface water.

Variable	Model 1: Physicochemical Model					Model 2: Physicochemical + Heavy metals Model				
	OR	Robust SE	p-value	Conf. Interval		OR	Robust SE	p-value	Conf. Interval	
pH	0.313	0.147	**0.013**	0.125	0.784	0.174	0.146	**0.037**	0.034	0.903
Electrical Conductivity	1.026	0.022	0.234	0.984	1.070	1.005	0.011	0.677	0.983	1.026
Total Dissolved solids	0.964	0.033	0.272	0.902	1.030	1.003	0.012	0.766	0.981	1.027
Turbidity	1.003	0.004	0.927	0.993	1.008	1.003	0.007	0.638	0.990	1.016
Total Suspended Solids	1.004	0.002	0.070	0.9996	1.009	1.005	0.003	0.120	0.999	1.012
Magnesium	0.997	0.095	0.979	0.828	1.202	0.913	0.123	0.500	0.701	1.189
Chloride	0.893	0.039	**0.010**	0.820	0.973	0.891	0.046	**0.025**	0.805	0.986
Phosphates	0.542	0.092	**<0.001**	0.388	0.757	0.452	0.135	**0.008**	0.252	0.812
Dissolved Oxygen	0.226	0.249	0.177	0.026	1.961	0.534	0.693	0.629	4.19E-02	6.801
Zinc						0.0001	0.001	**0.034**	3.27E-08	0.508
Copper						1.041	0.788	0.958	0.236	4.590
Nickel						0.325	0.429	0.394	0.024	4.320
Chromium						0.700	0.315	0.428	0.290	1.690

### Multivariate regression model of the relationship between faecal coliform, physicochemical and heavy metal concentrations in ground water

Table 6 is a nested model showing the multivariate relationship between faecal coliform, physicochemical and heavy metal concentrations in ground water. Higher values of total dissolved solids (OR = 0.940, p<0.05), turbidity (OR = 0.997, p<0.05) and phosphates (OR = 0.063, p<0.001) were associated with lower odds of detecting faecal coliform while higher values of electrical conductivity (OR = 1.042, p<0.05) and total suspended solids (OR = 1.023, p<0.05) were associated with higher odds of detecting faecal coliform.

**Table 6:** Multivariate negative log–log regression model predicting detection of faecal coliform in groundwater.

Variables	Model 1: Physicochemical Model					Model 2: Physicochemical + Heavy metals Model				
	OR	Robust SE	p-Value	Conf. Interval		OR	Robust SE	p-Value	Conf. Interval	
pH	0.491	0.397	0.380	0.101	2.399	0.387	0.419	0.381	0.046	3.233
Electrical Conductivity	1.042	0.016	**0.007**	1.011	1.073	1.097	0.036	**0.005**	1.029	1.170
Total Dissolved Solids	0.940	0.023	**0.012**	0.895	0.987	0.858	0.049	**0.007**	0.767	0.960
Turbidity	0.997	0.001	**0.016**	0.995	0.999	0.996	0.002	**0.025**	0.992	0.999
Total Suspended Solids	1.023	0.007	**0.001**	1.009	1.037	1.009	0.014	0.521	0.982	1.037
Magnesium	1.248	0.179	0.123	0.942	1.653	1.191	0.204	0.308	0.851	1.667
Nitrates	1.026	0.029	0.364	0.971	1.084	1.191	0.073	**0.004**	1.056	1.344
Phosphates	0.063	0.043	**<0.001**	0.017	0.239	0.043	0.036	**<0.001**	0.009	0.217
Chloride	1.042	0.045	0.348	0.957	1.134	1.047	0.123	0.696	0.831	1.319
Zinc						73.026	256	0.221	0.076	70609
Iron						2.627	3.984	0.524	0.134	51.321
Manganese						5.22E+12	1.64E+14	0.352	9.15E-15	2.98E+39
Nickel						6.09E-07	3.55E-06	**0.014**	6.74E-12	0.055
Chromium						0.174	0.165	0.066	0.027	1.120

Concentrations of heavy metals were controlled for in model 2. In this case, the relationship between electrical conductivity, total dissolved solids, turbidity and phosphates were robust and remained in predicting faecal coliform in ground water sources. The relationship between total suspended solids and detecting faecal coliform disappeared. However, a new relationship between nitrates (OR = 1.191, p<0.05) and detecting faecal coliform appeared, as it was in the bivariate model. Higher values of nitrates were associated with higher odds of detecting faecal coliform in ground water sources. Of all the heavy metals, only nickel (OR = 6.09E-07, p<0.05) was statistically significant in predicting faecal coliform. Higher values of nickel was associated with lower odds of detecting faecal coliform in ground water sources.

### Multivariate regression model of the relationship between total coliform, physicochemical and heavy metal concentrations in surface water

Table 7 is a nested model showing the multivariate relationship between total coliform, physicochemical and heavy metal concentrations in surface water. With the exception of magnesium (OR = 1.07, p<0.05), all physicochemical variables were not statistically significant in predicting total coliform in surface water sources. In this instance, higher values of magnesium were associated with the detection of total coliform in surface water sources.

**Table 7:** Multivariate negative log–log regression model predicting detection of total coliform in surface water.

Variables	Model 1: Physicochemical Model					Model 2: Physicochemical + Heavy metals Model				
	OR	Robust SE	p-value	Conf. Interval		OR	Robust SE	p-value	Conf. Interval	
pH	0.905	0.137	0.511	0.672	1.219	0.949	0.142	0.727	0.708	1.273
Electrical Conductivity	0.998	0.002	0.291	0.993	1.002	0.997	0.002	0.148	0.992	1.001
Total Dissolved Solids	1.005	0.004	0.166	0.998	1.012	1.006	0.003	0.096	0.999	1.012
Turbidity	1.000	0.005	0.933	0.999	1.001	1.000	0.002	0.809	0.997	1.003
Total Suspended Solids	0.998	0.001	0.159	0.996	1.001	0.998	0.002	0.325	0.995	1.002
Calcium	0.995	0.006	0.384	0.984	1.006	0.999	0.006	0.820	0.987	1.010
Phosphates	0.979	0.011	0.063	0.958	1.001	0.968	0.010	**0.002**	0.948	0.988
Magnesium	1.070	0.033	**0.030**	1.007	1.137	1.059	0.033	0.066	0.996	1.126
Zinc						2.429	1.809	0.233	0.565	10.451
Copper						0.902	0.189	0.623	0.599	1.360
Manganese						1.07E+07	1.22E+08	0.158	0.002	6.02E+16
Nickel						1.041	0.289	0.885	0.604	1.795
Chromium						0.876	0.161	0.471	0.610	1.256
Lead						3.95E-27	2.95E-25	0.415	1.26E-90	1.24E+37

Concentrations of heavy metals were controlled for in model 2. In this case, the relationship between magnesium was not robust and disappeared in predicting total coliform in surface water sources. A new relationship between phosphates (OR = 0.968, p<0.05) and detecting total coliform appeared. In this instance, higher values of phosphates was associated with lower odds of detecting total coliform. None of the heavy metals (zinc, copper, manganese, nickel and chromium) was statistically significant in predicting total coliform in surface water sources.

### Multivariate regression model of the relationship between total coliform, physicochemical and heavy metal concentrations in ground water

Table 8 is a nested model showing the multivariate relationship between total coliform, physicochemical and heavy metal concentrations in ground water. For the physicochemical parameters, higher values of pH (OR = 0.192, p<0.05) and phosphates (OR = 0.142, p<0.001) were associated with lower odds of detecting total coliform while higher values of total suspended solids (OR = 1.04, p<0.001) and nitrates (OR = 1.069, p<0.05) were associated with higher odds of detecting total coliform.

**Table 8:** Multivariate negative log–log regression model predicting detection of total coliform in ground water.

Variables	Model 1: Physicochemical Model					Model 2: Physicochemical + Heavy metals Model				
	OR	Robust SE	p-value	Conf. Interval		OR	Robust SE	p-value	Conf. Interval	
pH	0.192	0.119	**0.008**	0.057	0.648	0.083	0.064	**0.001**	0.018	0.376
Electrical Conductivity	1.030	0.039	0.441	0.956	1.110	1.060	0.053	0.240	0.962	1.168
Total Dissolved Solids	0.963	0.063	0.561	0.848	1.094	0.924	0.074	0.326	0.789	1.082
Turbidity	0.967	0.022	0.133	0.926	1.010	0.950	0.020	**0.014**	0.911	0.990
Total Suspended Solids	1.042	0.011	**<0.001**	1.020	1.065	1.054	0.018	**0.002**	1.019	1.090
Magnesium	0.933	0.073	0.376	0.800	1.088	0.821	0.094	0.086	0.655	1.029
Nitrates	1.069	0.028	**0.013**	1.014	1.126	1.074	0.041	0.060	0.997	1.157
Phosphates	0.142	0.070	**<0.001**	0.054	0.372	0.092	0.070	**0.002**	0.021	0.409
Chloride	0.899	0.053	0.073	0.801	1.010	0.860	0.056	**0.021**	0.757	0.978
Nickel						0.096	0.202	0.266	0.002	5.981
Chromium						0.776	0.757	0.795	0.115	5.245
Copper						2.569	1.475	0.100	0.833	7.918
Manganese						4463	63870	0.557	2.95E-09	6.76E+15
Iron						7.250	10.449	0.169	0.430	122
Zinc						0.047	0.187	0.442	1.97E-05	113.118

When heavy metals were controlled for in model 2, pH (OR = 0.083, p<0.05), total suspended solids (OR = 1.054, p<0.05) and phosphates (OR = 0.092, p<0.05) remained robust in predicting total coliform in ground water sources. The relationship between nitrates was not robust and disappeared in predicting total coliform in ground water sources. A new relationship between turbidity (OR = 0.950, p<0.05), chloride (OR = 0.860, p<0.05) and detecting total coliform appeared. In this instance, higher values of turbidity and chloride were associated with lower odds of detecting total coliform. None of the heavy metals (nickel, chromium, copper, manganese and iron) was statistically significant in predicting total coliform in ground water sources.

## Discussion

Overall, total suspended solids, magnesium, phosphates, chloride, dissolved oxygen, zinc, lead, copper, nickel and chromium were statistically significant predictors of faecal coliform in surface water at the bivariate level. At the multivariate level, only chloride, phosphates, pH and zinc were robust and persisted in predicting faecal coliform. This implied that higher values of chloride, phosphates, pH and zinc in surface waters could mean non-contamination by faecal coliform. Phosphate is a major nutrient for coliform bacteria [11, 12] and as such, phosphates’ significant relationship with faecal coliform at both bivariate and multivariate levels suggest its significance was not influenced by the presence of other water quality parameters. Also, the presence of chloride in water can be effective in suppressing the growth of bacteria and preventing the formation of biofilms hosting microorganism [13]. Karikari et al. [14] observed an inverse relationship with micro bacteria count including faecal coliform. Similarly, the robustness of zinc in predicting faecal coliform could suggest zinc is a major metallic component of surface water in this area. Bajjali [15] indicated that the availability of zinc is highly influenced by pH. The source of zinc could also be attributed to cation exchange from zinc bearing minerals at the soil-water interface, sewage and metallurgical waste. Also, pH is known to affect the survival of faecal coliform bacteria in water. Pearson et al. [16] reported that, faecal coliforms died more rapidly when pH conditions were increased above 8.50, even in a nutrient rich condition, suggesting the influence of pH for the survival of faecal coliform in water systems. The concentration and distribution of faecal coliform also depends on tropical conditions [17] and indirectly on the physicochemical conditions of the water [18].

In ground water sources, magnesium, nitrates, phosphates, chloride, lead, manganese and nickel were significant predictors of faecal coliform at the bivariate level. At the multivariate level, only electrical conductivity, total dissolved solids, turbidity, phosphates and nickel showed robustness in predicting faecal coliform. Total suspended solids and nitrates were completely mediated when heavy metals were accounted for in the model. In this instance, higher values of phosphates, total dissolved solids, turbidity and nickel in groundwater meant non-contamination by faecal coliform. However, higher values of electrical conductivity, total suspended solids and nitrates meant faecal coliform contamination of groundwater sources. The survival of faecal coliform is affected by several factors including salinity and metal toxicity [19, 20]. Saline waters tend to reduce the survival of coliform bacteria. A saline water is expected to contain dissolved salts, especially for ground water where the dissolution of salt from minerals and rocks are common. Electrical conductivity depends on the availability of soluble salts in water and as such, the relationship between conductivity and faecal coliform could be mediated by total dissolved solids. The survival of coliform bacteria could as well be hindered by the presence of heavy metals in water. Mallin et al. [21] found that faecal coliform densities were strongly correlated with turbidity (positively), however, in this study, turbidity of groundwater relates to non-contamination by faecal coliform. The sources of turbidity could emanate from clay particles as well as weathering processes.

The availability of suspended particles in the form of mineral precipitates and organic matter could provide protection for coliform bacteria. Coliform bacteria could be attached to clay through adsorption and sedimentation [22, 23]. Metal colloids, organic materials and clay minerals such as montmorillonite could provide these protections by forming protective envelopes around them [24]. Precipitation of metals could contribute to total suspended solids and as such influence its relationship with faecal coliform. Heavy metals have been found to be associated with sludge solids [25]. Suspended solids could also provide coliform bacteria with organic and inorganic nutrients [20]. The concentrations of faecal coliform have been reported in many studies to be associated with phosphorus and nitrogen concentrations [26–28]. However, in this study, high phosphates were found to be associated with low concentrations of faecal coliform, and this could suggest that faecal coliforms feed on phosphates more than nitrogen and as such high concentrations of phosphates could mean low or absence of faecal coliform bacteria. The mediation of nitrates by heavy metals further suggest its association with faecal coliform is not strong and this occurred only in ground water both at the bivariate and multivariate levels where dissolution of metal is expected to be much greater.

Coliform bacteria levels in water is also affected by the complex interactions between biological, physical and chemical parameters. These interactions are controlled by varying factors in different aquatic environments. In surface water, faecal coliform survival and concentration is much affected by physical and climatic factors such as water temperature, rainfall, runoff, tidal conditions, solar radiation, dissolved nutrients, competition with other bacteria as well as other physicochemical conditions [17–19, 28, 29]. Geochemical processes like weathering, dissolution, hydrolysis, precipitation, adsorption, and ion exchange as well as oxidation reduction and biochemical reactions are major controlling factors for the chemistry of groundwater [30]. Other factors such as temperature, dissolution and physicochemical conditions could be common in both systems, however, their variations differ. Temperature variation in surface water is much greater than ground water; ground water temperature is much steadier. Other factors such as surface runoff and solar radiation might not affect ground water systems. Surface water pH is much influenced by other factors such as dissolved organic matter and carbon dioxide. Principal component analysis by [18] indicated that, faecal coliform was associated with external sources such as precipitation and runoff. These external factors directly influence surface water than ground water. Thus, the survival and association of faecal coliform with physicochemical parameters and heavy metals is expected to exhibit varying relationships in different aquatic environments.

For total coliform in surface water systems, only magnesium and manganese were statistically significant in predicting total coliform at the bivariate level. At the multivariate level, only magnesium and phosphates predicted total coliform although the predictive associations were not robust. In this case, higher values of magnesium implied total coliform contamination while higher values of phosphates implied non-contamination by total coliform. The presence of phosphates serves as nutrient for coliforms. Lim et al. [31] observed an increase in *Escherichia coli* concentration when orthophosphates were added as a source of phosphate in lake water. This is an indication that coliform bacteria feed on phosphates. High levels of phosphates could relate to the absence or low levels of coliform. The mediation of phosphates when heavy metals were accounted for, suggest that heavy metals might control phosphates’ relationship with total coliform in surface water and thus the sources of phosphates in surface water could be linked to bedrock phosphate mineralization, metallurgical wastewater, as well as, weathering processes. Coliform bacteria levels in water bodies are known to be affected by salinity and temperature and closely related with biological conditions and physicochemical properties of the water including pH, temperature, suspended solids, organic and inorganic nutrients in water [17, 32].

For ground water sources at the bivariate level, magnesium, nitrates, phosphates, chloride, cadmium, lead and nickel were statistically significant predictors of total coliform. At the multivariate level, only pH, total suspended solids and phosphates were robust in predicting total coliform in ground water systems. The predictive association of turbidity, chloride and nitrates were mediated by heavy metals. Higher values of pH, phosphates, turbidity and chloride in ground water implied non-contamination by total coliform. However, higher values of total suspended solids and nitrates meant contamination of total coliform in ground water sources. Phosphates showed its association with total coliform in groundwater just as it was observed for faecal coliform. Phosphates association with faecal and total coliform further suggest its contribution to the survival of coliform bacteria as a major source of nutrient for bacteria in groundwater. The pH values for surface water in the study area ranged from highly acidic to slightly alkaline as compared to slightly acidic to neutral for ground water. The pH values outside the neutral pH conditions for surface water might not favor the survival of faecal coliform bacteria and this reflected in relatively low faecal coliform count for surface water. This was also reported in other studies [18, 30, 33, 34]. Total coliform count was still high for surface water, suggesting other bacteria groups of coliforms might withstand this pH condition. A similar observation was reported by [27, 35, 36] where total coliform bacteria concentrations were higher than faecal coliform concentration in river samples.

Total suspended solids were associated with both faecal and total coliforms in predicting coliform contamination. The presence of suspended solids can be related to the survival of coliform bacteria. Suspended solids promote coliform survival by protecting them from adverse conditions such as metal toxicity, UV radiations and predations [37, 38] and could also provide nutrients for coliform bacteria [20]. Microorganisms that are attached to suspended particles can withstand hostile environmental conditions better than their free-living counterparts [39]. However, in this study, total suspended solids association with faecal coliform was affected by the introduction of heavy metals while it association with total coliform was robust. Total coliform comprises of several different species of bacteria such as nitrifying bacteria and other types and each of these has its own resistance to adverse conditions. Anderson et al. [22] observed a varying response by different coliform bacteria to stress in aquatic environment. This diversity in resistance gives other constituents of total coliform bacteria a slight advantage in surviving such hostile environmental conditions. These different bacteria constituents of total coliform could also be competing with faecal coliform for survival in such conditions [39].

Bacteria contamination of surface water in this study area could be more of faecal origin than in groundwater. This showed in faecal coliform’s conspicuous association with water quality parameters in surface water as compared to groundwater. Faecal coliform could also face competition from other micro bacteria for limited nutrient and survival in ground water. The mediation of major ions (nitrates, phosphates, chloride and magnesium) by heavy metals suggests the presence of soluble salt in groundwater is controlled by mineralization. Total coliform shows much more association with water quality parameters in ground water than in surface water. Local authorities need to monitor, manage and remediate surface and ground water sources for potential disease causing microbes in ways that takes into consideration the factors that create different conditions in the two water systems.

## Conclusion

This study assessed the relationship between coliform bacteria and water geochemistry in surface and ground water systems in the Tarkwa mining area using logistic regressions models. Bacteria contamination of surface water in this study area could be more of faecal origin than in groundwater. This showed in faecal coliform’s conspicuous association with water quality parameters in surface water as compared to groundwater. Faecal coliform could also face competition from other micro bacteria for limited nutrient and survival in ground water. The mediation of major ions (nitrates, phosphates, chloride and magnesium) by heavy metals suggests the presence of soluble salt in groundwater is controlled by mineralization. Total coliform shows much more association with water quality parameters in ground water than in surface water. This study establishes the need to monitor, manage and remediate surface and ground water sources for potential disease causing microbes in ways that takes into consideration the factors that create different conditions in the two water systems. This study validates the usefulness of statistical models as tools for preventing surface and ground water contamination.
